# *Lactococcus* G423 improve growth performance and lipid metabolism of broilers through modulating the gut microbiota and metabolites

**DOI:** 10.3389/fmicb.2024.1381756

**Published:** 2024-06-13

**Authors:** Mi Wang, Wei Ma, Chunqiang Wang, Desheng Li

**Affiliations:** College of Animal Husbandry and Veterinary Medicine, Jinzhou Medical University, Jinzhou, China

**Keywords:** *Lactococcus*, broilers, growth performant, 16S rRNA, LC–MS

## Abstract

This study aimed to explore whether *Lactococcus* G423 could improve growth performance and lipid metabolism of broilers by the modulation of gut microbiota and metabolites. A total of 640 1-day-old AA broilers were randomly divided into 4 groups [Control (CON), Lac_L, Lac_H, and ABX]. Average daily gain (ADG), average daily feed intake (ADFI), feed conversion ratio (FCR), breast muscle, thigh muscle, and abdominal fat pad were removed and weighed at 42 days of age. Serum was obtained by centrifuging blood sample from jugular vein (10 mL) for determining high-density lipoprotein (HDL), total cholesterol (TC), low-density lipoprotein (LDL), and triglyceride (TG) using ELISA. The ileal contents were harvested and immediately frozen in liquid nitrogen for 16S rRNA and LC–MS analyses. Then, the results of 16S rRNA analysis were confirmed by quantitative polymerase chain reaction (qPCR). Compared with the CON group, FCR significantly decreased in the Lac_H group (*p* < 0.05) in 1–21 days; ADG significantly increased and FCR significantly decreased in the Lac_H group (*p* < 0.05) in 22–42 days. 42 days weight body and ADG significantly increased in the Lac_H group (*p* < 0.05) in 42 days. Abdominal fat percentage was significantly decreased by *Lactococcus* G423 (*p* < 0.05), the high dose of *Lactococcus* G423 significantly decreased the serum of TG, TC, and LDL level (*p* < 0.05), and the low dose of *Lactococcus* G423 significantly decreased the serum of TG and TC level (*p* < 0.05). A significant difference in microbial diversity was found among the four groups. Compared with the CON group, the abundance rates of *Firmicutes and Lactobacillus* in the Lac_H group were significantly increased (*p <* 0.05). The global and overview maps and membrane transport in the Lac_L, Lac_H, and ABX groups significantly changed versus those in the CON group (*p* < 0.05). The results of LC–MS demonstrated that *Lactococcus* could significantly improve the levels of some metabolites (6-hydroxy-5-methoxyindole glucuronide, 9,10-DiHOME, *N*-Acetyl-l-phenylalanine, and kynurenine), and these metabolites were involved in four metabolic pathways. Among them, the pathways of linoleic acid metabolism, phenylalanine metabolism, and pentose and glucuronate interconversions significantly changed (*p* < 0.05). *Lactococcus* G423 could ameliorate growth performance and lipid metabolism of broilers by the modulation of gut microbiota and metabolites.

## 1 Introduction

Intestinal microbes and host are bioactive communities, forming the junction between animals and their nutritional environment (Anand and Mande, 2018). Thus, microbiota may affect the physiology and metabolism of host, and certain healthy bacteria in the add microbiota may improve gut health (Judkins et al., 2020; Ghosh et al., 2021; Gill et al., 2021). Intestinal microbes have noticeably attracted researchers’ attention recently. Over the past two decades, some studies revealed that antibiotics can alter the likely benefit of the host–microbiota interaction or relationship by regulating the microbiota (Yukgehnaish et al., 2020). In the poultry industry, antibiotics have been widely used (Angela et al., 2020). However, it is essential to pay further attention to antibiotic resistance (Hakimul et al., 2020; Paintsil et al., 2021), and long-term use of these antibiotics could cause antibiotic residues remaining in animals, in which they seriously threaten human health (Dawood et al., 2018). It is well known that the basic function of probiotics is to reduce gut-related diseases by regulating and improving the intestinal microbial balance in humans (Soccol et al., 2010; Zommiti et al., 2020). Recently, probiotics have been found to benefit not only human health but also animal health (Shi et al., 2020). Several studies demonstrated that beneficial effects of probiotics for the host included suppression of growth of pathogens, modulation of the immune system, improvement of nutrient metabolism, and modification of the composition of the intestinal microbiota (Kailasapathy and Chin, 2000; Chan and Zhang, 2005; Ashouri et al., 2020; Fan et al., 2021). Especially, an appropriate amount of *lactic acid bacteria* (LAB) can regulate the microflora in the gut (Kim et al., 2021). Some studies show that LAB could significantly improve lipid metabolism and fat deposition (Cho et al., 2020; Wang et al., 2023). Zhang et al. (2022) report that LAB have an effect on production performance, lipid metabolism, and meat quality in heat–stressed broilers. Previous studies have shown that LAB can increase the bacterial phylogenetic diversity in the gut of mice (Usui et al., 2018) and weaning piglets (Zhao et al., 2016). Gupta et al. (2018) also report that LAB can modulate the composition and interaction of the intestinal microbiota of Atlantic salmon. *Lactococcus* is industrially crucial *LAB* used to produce lactic acid, pickled vegetables, buttermilk, cheese, and several types of dairy foods and drinks. In addition, they are utilized as probiotics in specific formulations. *Lactococcus* can modulate intestinal microbiota of animals (Busti et al., 2020; Tan et al., 2022). *Lactococcus lactis* has the potential to enhance growth performance, immune function, and intestinal development in broiler chickens (Zhou et al., 2019). Zhang et al. (2016) also study showed that *Lactococcus* could enhance the growth performance of broiler chickens and improve their health. However, they have rarely been studied versus other *LAB* genera. The ribosomal RNA (16S) rRNA (16S rRNA) gene possesses the advantage of exploring the composition of the gut microbiota of chickens (Shang et al., 2018), broiler chickens (Mohd Shaufi et al., 2015), Dagu chickens (Xu et al., 2016), and naked neck chickens (Park et al., 2016). Liquid chromatography-mass spectrometry (LC–MS) has solid analytical capability, and it can detect the association of bacteria and metabolites with high resolution and accuracy (Xia et al., 2021). Moreover, correlation analysis between microorganisms and metabolites was performed. This was of great significance in revealing the contribution of *Lactococcus* G423 to the formation of metabolites in the gut. Therefore, the present study aimed to explore whether *Lactococcus* G423 could ameliorate growth performance and lipid metabolism of broilers by the modulation of gut microbiota and metabolites.

## 2 Materials and methods

### 2.1 Birds, diets, and experimental design

Totally, 640 1-day-old AA broilers (Shu-ya Poultry Co., Ltd., Tieling, China) were randomly classified into four experimental groups, and each group included 160 birds (8 replicates of 20 birds). Birds were raised in stainless steel cages (400 mm × 450 mm × 1,500 mm) in a controlled room for 42 days. This study was performed at Poultry Research Farm, Jinzhou Medical University, Liaoning, China. The temperature of room was gradually reduced by 3.0–3.5°C weekly until achieving a thermo-neutral zone ranged from 21 to 26°C by the end of the 3rd week. The experimental diets were based on corn and soybean meal. Four dietary regimes were provided as follows: control group (basal diet, CON group), Lac-L and Lac-H groups (basal diet supplemented with 50 and 100 mg/kg *Lactococcus* G423, respectively), and ABX group (basal diet supplemented with 50 mg/kg narasin). The basal diet was divided into two phases: the starter phase from 1 to 21 days and the growth phase from 21 to 42 days. The basal diet was formulated to meet the nutritional requirements according to the Chinese Broiler Feeding Standards (NY/T33-2004) (Table 1). *Lactococcus*G423 (1 × 10^10^ CFU/g) and narasin (purity of narasin dihydrate powder 15%, Eli Lilly and Company, Indianapolis, Indiana, United States) were mixed in basal diet (Supplementary Figure S1).

**Table 1:** Calculated composition of basal diets and nutrient levels.

	% (air-dry basis)	
Composition	1–21 days	21–42 days
Ingredients		
Corn	57.50	62.22
Soybean meal	30.50	29.00
Corn gluten meal	5.00	1.00
Soybean oil	3.00	4.00
Sodium chloride	0.30	0.30
Dicalcium phosphate	1.65	1.70
Limestone	1.52	1.23
Methionine	0.25	0.20
Choline	0.15	0.15
Multivitamin premix^a^	0.03	0.03
Mineral premix^b^	0.10	0.10
Nutrient level		
Metabolizable energy (MJ/kg)	12.33	12.50
Crude protein	21.75	19.72
Lysine	1.18	1.04
Methionine + Cysteine	0.91	0.86
Ca	1.07	0.60
Total *P*	0.70	0.68
Available *P*	0.46	0.45

^a^Content per kilogram of diet: 1,750 IU of vitamin A; 3,500 IU of vitamin D3; 12 IU of vitamin E, 0.7 mg of vitamin K, 1.9 mg of vitamin B1, 3.8 mg of vitamin B2, 3.7 mg of vitamin B6, 0.02 mg of vitamin B12, 0.18 mg of biotin, 0.57 mg of folic acid, 33 mg of niacin, and 13 mg of pantothenic acid. ^b^Content: 8 mg of Cu (CuSO_4_·5H_2_O), 0.35 mg of I (KI), 80 mg of Fe (FeSO_4_·7H_2_O), 60 mg of Mn (MnSO_4_·H_2_O), 0.15 mg of Se (NaSeO_3_), and 40 mg of Zn (ZnO).

### 2.2 Growth and carcass measurements

Broiler performance in terms of average daily gain (ADG), average daily feed intake (ADFI), survival rate, and feed conversion ratio (FCR) was weekly recorded, in which ADG, ADFI, and FCR were calculated and presented for 6-week experimental period. Breast muscle, thigh muscle, and abdominal fat pad (including fat surrounding the gizzard, bursa of Fabricius, cloaca, and adjacent muscles) from one bird of average BW per replicate were removed and weighed at week 6. To compensate for the differences in carcass weight, these values were expressed as a percentage of carcass weight.

### 2.3 Enzyme-linked immunosorbent assay

Content of high-density lipoprotein (HDL), LDL, TG, and TC was determined using enzyme-labeled instrument according to ELISA kit instruction (Nanjing Jiancheng Bio. Institute, Nanjing, Jiangsu, China).

### 2.4 Illumina MiSeq sequencing for the detection of intestinal microbial diversity

Eight ileal samples per group were randomly selected for the analysis of intestinal flora. The polymerase chain reaction (PCR) amplification of the hypervariable region V3–V4 of the 16S rRNA gene was performed with the universal primers set338 F (5′-ACTCCTACGGAGGCAGCAG-3′) and 806R (5′-GGACTACHVGGGTWTC TAAT-3′) (Liu et al., 2016). The quality and concentration of DNA were determined by 1.0% agarose gel electrophoresis and a NanoDrop^®^ ND-2000 spectrophotometer (Thermo Fisher Scientific Inc., Waltham, MA, United States) and kept at −80°C for further experiment. All samples were amplified in triplicate. The PCR products were extracted from 2% agarose gel and were purified using the AxyPrep DNA Gel Extraction Kit (Axygen Biosciences, Union City, CA, United States), according to the manufacturer’s instructions and were quantified using Quantus™ Fluorometer (Promega, Madison, WI, United States). The Illumina MiSeq platform (Illumina Inc., San Diego, CA, United States) was used for paired-end sequencing (2 × 300) of the PCR products. The raw sequence reads were quality-filtered and merged by FLASH (Tanja and Steven, 2011) before open-reference operational taxonomic unit (OTU) picking via UPARSE (Stackebrandt and Goebel, 1994; Edgar, 2013) and taxonomy classification through the SILVA 16S rRNA database (Wang, 2007).

### 2.5 Quantitative PCR (qPCR)

*Lactobacillus* and *Firmicutes* were detected by qPCR. Eight ileum contents from broiler were collected. The primers used for qPCR are presented in Table 2. The conditions of PCR reaction were summarized as follows: (1) at 95°C for 5 min; (2) a: at 95°C for 30 s; b: at 60°C for 30 s; c: at 72°C for 1 min, including 35 cycles; (3) a: at 95°C for 30 s; b: at 55°C for 30 s; c: at 72°C for 1 min. The ΔC*t* was calculated as follows: (corrected sample) = mean value of target gene–mean value of internal reference gene (ΔΔ Ct = ΔCt–mean value of control group).

**Table 2:** Primers used for qPCR.

Gene	Primer sequence	Product size
*Firmicutes*	F:5′- GGAGYATGTGGTTTAATTCGAAGCA-3′	200 bp
	R: 5′-AGCTGACGACAACCATGCAC-3′	
*Lactobacillus*	F: 5′-AGCAGTAGGGAATCTTCCA-3′	340 bp
	R: 5′-ATTYCACCGCTACACATG-3′	
18sRNA	F:5′-TAGATAACCTCGAGCCGATCGCA-3′	312 bp
	R:5′-GACTTGCCCTCCAATGGATCC TC-3′	

### 2.6 LC–MS analysis

Eight ileal samples Con and Lac_H group were randomly selected for the analysis of LC–MS. The LC–MS analysis of ileal contents was conducted on a Thermo UHPLC-Q Exactive HF-X system equipped with an ACQUITY HSS T3 column (100 mm × 2.1 mm i.d., 1.8 μm; Waters Corp., Milford, MA, United States) at Majorbio Bio-Pharm Technology Co., Ltd. (Shanghai, China). The mass spectrometric data were collected using a Thermo UHPLC-Q Exactive HF-X Mass spectrometer equipped with an electrospray ionization (ESI) source operating in positive and negative modes. The pretreatment of LC–MS raw data was performed by Progenesis QI software (Waters Corp.), and a three-dimensional (3D) data matrix in CSV format was exported. This 3D matrix included the following information: sample information, metabolite name, and mass spectral response intensity. Internal standard peaks and any known false positive peaks (including noise, column bleed, and derivatized reagent peaks) were removed from the data matrix, de-redundant, and peak pooled. Moreover, the metabolites were identified by searching in the following databases: Human Metabolome Database (HMDB)^1^, Metlin^2^, and Majorbio^3^ (Kong et al., 2022; Li C. et al., 2022; Li Z. et al., 2022).

### 2.7 Statistical analysis

Between-group statistical differences were compared using one-way analysis of variance (ANOVA), followed by post-hoc multiple comparisons using Fisher’s least significant difference (LSD) *t*-test. The experimental data were presented as the mean ± standard error of the mean (SEM), which were analyzed using SPSS 20.0 software (IBM, Armonk, NY, United States), and *p* < 0.05 was considered statistically significant. The 16S rRNA genes of gut microbiota were analyzed using an online platform (see Footnote 3) (Ren et al., 2022). The multivariate statistical analysis was performed using the “ropls” (version 1.6.2) R package from Bioconductor on Majorbio Cloud Platform (see Footnote 3) (Ren et al., 2022).

## 3 Results

### 3.1 Growth performance

Compared with control, FCR significantly decreased in the Lac_H group (*p* < 0.05) in 1–21d; ADG significantly increased and FCR significantly decreased in the Lac_H group (*p* < 0.05) in 22–42 days; weight body and ADG significantly increased in the Lac_H group (*p* < 0.05) in 42 days. There were no significant changes in FCR, survival rate, and ADFI (*p* > 0.05) among Lac_H, Lac_L, and ABX groups in 42 days (Table 3).

**Table 3:** Effects of *Lactococcus* on the growth performance in broilers.

	42 days			
	CON	Lac_L	Lac_H	ABX
1–21 days				
21 days weight body (g)	911 ± 5.13	919 ± 4.94	919 ± 4.88	903 ± 5.40
ADG (g)	41.5 ± 0.24	41.9 ± 0.23	41.8 ± 0.23	41.1 ± 0.26
ADFI (g)	50.1 ± 0.09	50.7 ± 0.26	49.6 ± 0.31	49.7 ± 0.29
Survival rate (%)	97.5 ± 1.02	97.5 ± 1.03	98.1 ± 1.19	98.7 ± 0.94
FCR	1.15 ± 0.009^a^	1.15 ± 0.004^ac^	1.12 ± 0.005^bc^	1.14 ± 0.007^a^
22–42 days				
ADG (g)	74.9 ± 1.58^a^	76.8 ± 2.26^ab^	81.8 ± 1.80^b^	77.1 ± 1.27^ab^
ADFI (g)	125.2 ± 0.93	129.3 ± 1.58	127.7 ± 1.51	126.6 ± 1.21
FCR	1.67 ± 0.02^a^	1.68 ± 0.03^a^	1.56 ± 0.04^b^	1.64 ± 0.02^ab^
42 days				
42 days weight body (g)	2,485 ± 36.4^a^	2,532 ± 44.7^ab^	2,637 ± 40.8^b^	2,523 ± 35.7^ab^
ADG (g)	59.17 ± 0.87^a^	60.30 ± 1.07^a^	62.80 ± 0.96^b^	60.08 ± 0.75^a^
ADFI (g)	88.99 ± 0.77	88.28 ± 0.75	91.59 ± 3.14	88.27 ± 0.95
Survival rate (%)	94.75 ± 1.18	97.75 ± 1.03	97.5 ± 1.77	95.6 ± 1.13
FCR	1.50 ± 0.028	1.47 ± 0.018	1.46 ± 0.022	1.47 ± 0.014

In the same row, values with different superscripts represent significant differences (*p* < 0.05). Values are expressed as mean ± SEM (*n* = 8 for all groups).

### 3.2 Carcass characteristics

Compared with control, abdominal fat percentage was significantly decreased by *Lactococcus* G423 (*p* < 0.05); however, dressing percentage, thigh muscle percentage, and breast muscle percentage had no significant changes among Lac_H, Lac_L, and ABX groups (*p* > 0.05) (Table 4).

**Table 4:** Effect of *Lactococcus* on carcass characteristic in broilers.

Items	Control	Lac_L	Lac_H	ABX
Dressing percentage (%)	92.28 ± 0.27	92.18 ± 0.41	91.35 ± 0.44	91.93 ± 0.02
Breast muscle (%)	30.04 ± 0.48	31.08 ± 1.70	32.28 ± 0.42	30.24 ± 0.88
Thigh muscle (%)	31.61 ± 0.56	32.04 ± 0.70	32.19 ± 0.56	32.10 ± 1.98
Abdominal fat (%)	2.51 ± 0.11^a^	1.65 ± 0.11^b^	1.04 ± 0.16^b^	2.19 ± 0.14^a^

In the same row, values with different superscripts represent significant differences (*p* < 0.05). Values are expressed as mean ± SEM, and *n* = 8 for all groups.

### 3.3 Serum biochemical parameters

**Figure 1:**
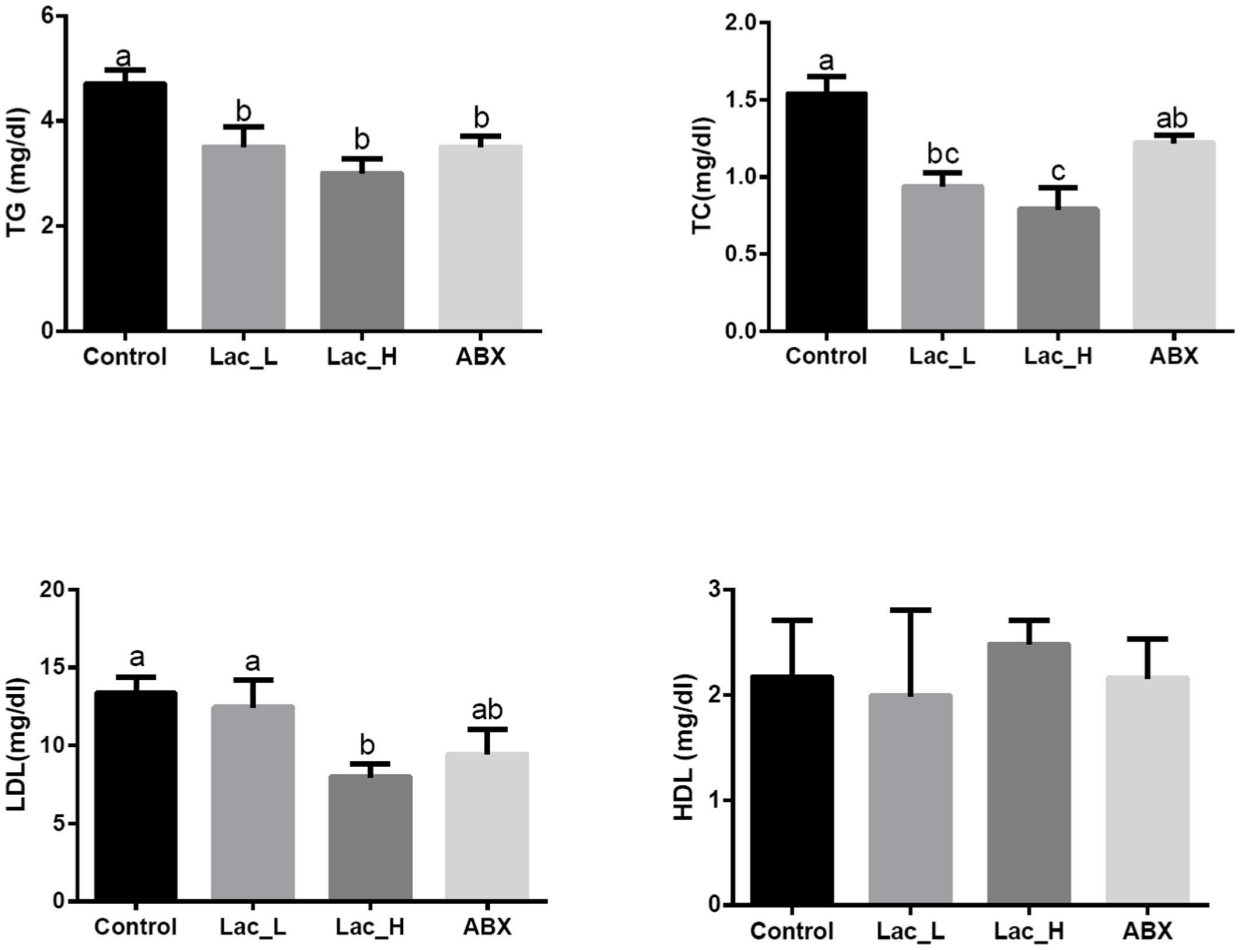
Effect of *Lactococcus* on serum biochemical parameters in broilers. Values with different superscripts represent significant differences (*p* < 0.05).

Comparing with control, the high dose of *Lactococcus* G423 significantly decreased the serum of TG, TC, and LDL level (*p* < 0.05), and the low dose of *Lactococcus* G423 significantly decreased the serum of TG and TC level (*p* < 0.05). ABX significantly decreased the content of TG (*p* < 0.05) in serum; however, HDL content had no significant changes among Lac_H, Lac_L, and ABX groups (*p* > 0.05) (Figure 1).

### 3.4 Intestinal microflora

To characterize the intestinal microbiota composition of broilers in the four groups, 16S rRNA gene sequence analysis was performed. With the sequence similarity of 97%, 903 OTUs were obtained. The average good’s coverage for samples was higher than 99%, indicating that the majority of the microbial species were identified. and sequencing depth was also adequate for the robust sequence analysis.

As shown in Table 5, alpha diversity analysis of gut microbiota showed that compared with the CON group, the Chao and Ace indices in the Lac_H, Lac_L, and ABX groups significantly increased (*p* < 0.05); however, the Simpson index exhibited an opposite trend. The Simpson index in the Lac_L, Lac_H, and ABX groups was significantly reduced compared with that in the CON group (*p* < 0.05). In addition, the Sob index in the Lac_H and ABX groups was significantly higher than that in the CON group (*p* < 0.05). The Shannon index in the Lac_L and Lac_H groups was significantly elevated compared with that in the CON group (*p* < 0.05). The Coverage index in the Lac_L and ABX groups significantly increased compared with that in the CON group (*p* < 0.05). The effects of *Lactococcus* on the diversity and richness of intestinal microbiota community in broilers were evaluated based on alpha diversity (Table 5).

**Table 5:** Alpha diversity of intestinal microbiota based on OTU levels.

Index	CON	Lac_L	Lac_H	ABX
Coverage	0.99986 ± 0.00011^a^	0.99914 ± 0.00013^b^	0.99934 ± 0.00043^a^	0.99929 ± 0.00027^b^
Sobs	15.00 ± 4.000^a^	120.00 ± 65.483^a^	230.33 ± 69.601^b^	177.33 ± 82.008^b^
Shannon	0.7808 ± 0.11812^a^	1.2905 ± 0.20873^b^	2.3078 ± 0.84541^b^	2.0740 ± 1.08200^ab^
Simpson	0.60533 ± 0.06405^a^	0.40857 ± 0.07641^b^	0.20462 ± 0.08412^c^	0.24308 ± 0.13222^bc^
Ace	44.60 ± 28.67^a^	173.16 ± 50.96^b^	253.89 ± 75.98^b^	197.65 ± 75.66^b^
Chao	32.00 ± 22.07^a^	154.38 ± 68.31^b^	255.83 ± 79.42^b^	201.12 ± 76.12^b^

In the same row, values with different superscripts indicate significant differences (*p* < 0.05). Values are expressed as mean ± SEM (*n* = 8 for all groups).

**Figure 2:**
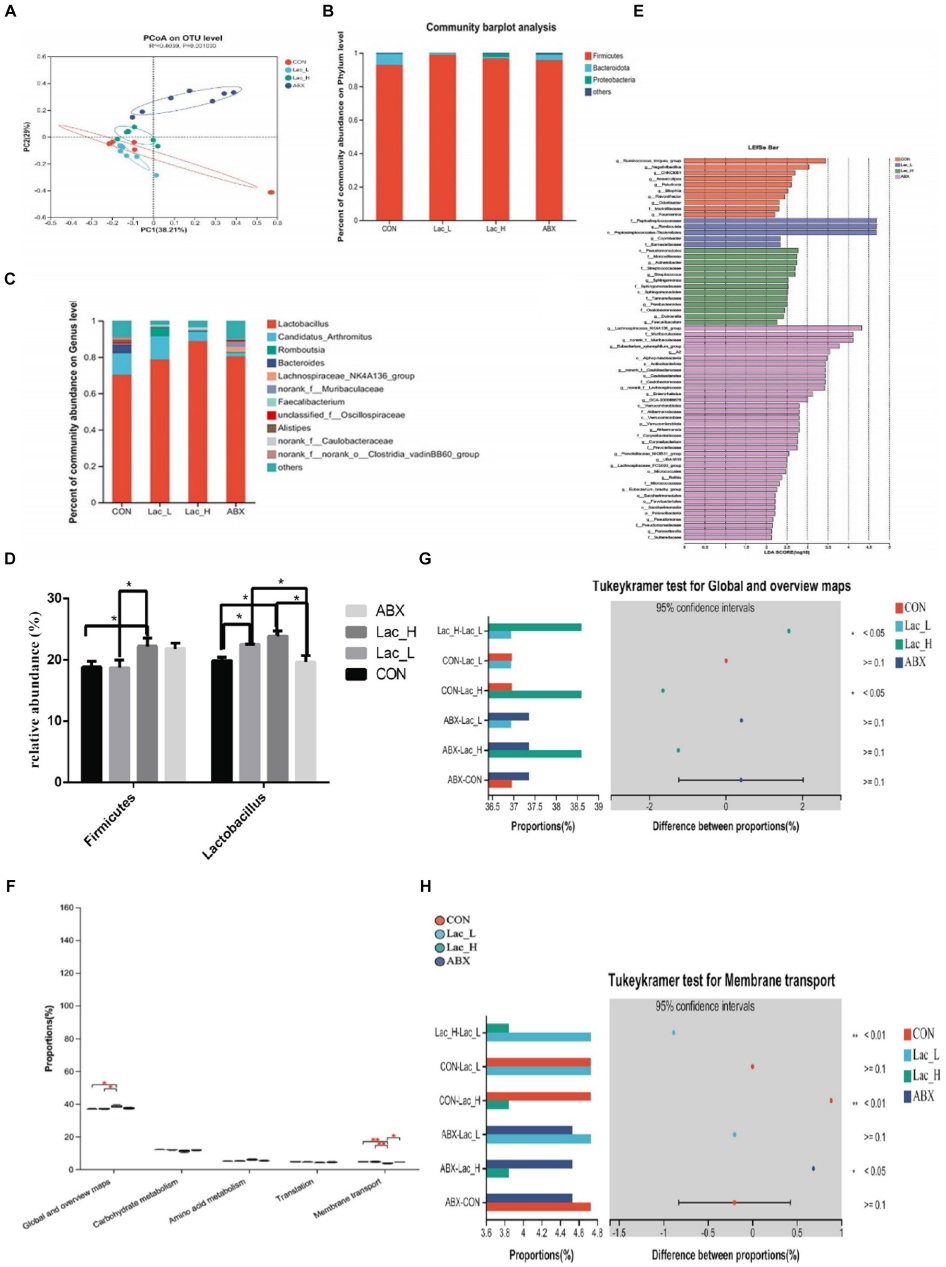
Effects of *Lactococci* on gut microbiota of broilers. Principal coordinate analysis (PCoA) based on the weighted UniFrac distance **(A)**; column chart of community difference at the phylum level **(B)** and the genus level **(C)**; relative abundance of discriminative gut microbiota at the genus level **(D)**; LEfSe analysis **(E)**; KEGG pathway analysis **(F–H)**; ^*^ and ^**^ represent *p* < 0.05 and *p* < 0.01, respectively.

Based on OTU abundance, principal coordinate analysis (PCoA) showed that points in the Lac_H and ABX groups were scattered in the right, which indicated that the microbial structure in the Lac_H and ABX groups had undergone a tremendous change versus that in the CON group. In the Lac_L and CON groups, points were clustered separately from each other in the left, which showed that the low dose of *Lactobacillus* G423 could change the structure of gut microbiota (Figure 2A). At the phylum level, *Firmicutes*, *Proteobacteria*, and *Bacteroidetes* were the most of species identified in all samples (Figure 2B). At the genus level, compared with those in the CON group, the abundance of *Lactobacillus* was higher, whereas that of *Bacteroides* was lower in the Lac_L,Lac_H and ABX groups (Figure 2C). As shown in Figure 2D, qPCR showed that the proportion of *Lactobacillus* in the Lac_L and Lac_H groups was significantly elevated compared with that in the CON and ABX groups (*p* < 0.05). Additionally, the proportion of *Firmicutes* was significantly risen after treating with Lac_H (*p* < 0.05). The different effects of Lac_L and Lac_H on microbiota might justify their different number of microorganisms. Subsequent linear discriminant analysis effect size (LEfSe) revealed substantial differences in *Lactobacillus_salivarius* and *Lactobacillus_johnsonii* in the Lac_H and ABX groups (Figure 2E).

The function of the ileum microbiome was predicted using the phylogenetic investigation of communities by the reconstruction of unobserved species 2 (PICRUSt2). Then, the Kyoto Encyclopedia of Genes and Genomes (KEGG) pathway analysis was used to divide the predicted metabolic pathways into six functional groups. The microbial communities in the CON, Lac_L, Lac_H, and ABX groups were mainly related to metabolism, genetic information processing, cellular processes, environmental information processing, human diseases, and organic systems. Their main functions were concentrated in the metabolism of amino acids, carbohydrate, vitamins, terpenoids, polyketides, and lipids (Table 5).

As shown in Figure 2F, the global and overview maps and membrane transport in the Lac_L, Lac_H, and ABX groups significantly changed compared with those in the CON group (*p* < 0.05). Functional predictions of differences in the mean relative abundance among groups are shown in Figures 2G,H and Table 6.

**Table 6:** Functional prediction of colonic microbiota in broilers.

Pathway level1	Pathway level2	ABX	CON	Lac_H	Lac_L
Metabolism	Global and overview maps	25453165	25209771	32812686	22747869
Metabolism	Carbohydrate metabolism	8027842	8296218	9459116	7362105
Metabolism	Amino acid metabolism	3727261	3505550	5108916	3255079
Metabolism	Energy metabolism	2772159	2959623	3509201	2687047
Metabolism	Nucleotide metabolism	2337797	2471719	2856356	2138305
Metabolism	Metabolism of cofactors and vitamins	2118528	1949155	2856380	1844675
Metabolism	Lipid metabolism	1660032	1863905	2096791	1617754
Metabolism	Metabolism of other amino acids	1042572	1039656	1262152	915605.6
Metabolism	Glycan biosynthesis and metabolism	956051.8	949022.1	1256285	841718.6
Metabolism	Biosynthesis of other secondary metabolites	831607.5	750127.1	1141571	696283.5
Metabolism	Xenobiotics biodegradation and metabolism	788123.3	775451.7	965045.2	680400.6
Metabolism	Metabolism of terpenoids and polyketides	697175.4	606856.4	837099.5	537757.8
Genetic Information Processing	Translation	3066636	3226340	3675862	2842519
Genetic Information Processing	Replication and repair	2707168	2879987	3182682	2538806
Genetic Information Processing	Folding, sorting and degradation	1085844	1068083	1308536	971930.5
Genetic Information Processing	Transcription	179590.6	196478.2	205977.1	168491.3
Environmental Information Processing	Membrane transport	3083471	3210148	3247480	2901880
Environmental Information Processing	Signal transduction	1800463	1766267	2086185	1568141
Environmental Information Processing	Signaling molecules and interaction	6.32	0.632	0.632	0.632
Cellular Processes	Cellular community – prokaryotes	1378514	1479321	1616860	1391040
Cellular Processes	Cell growth and death	504428.8	478711.9	639126.7	436497.3
Cellular Processes	Cell motility	267078	7688.8	270773.6	146903.1
Cellular Processes	Transport and catabolism	77755.92	52705.91	111201.6	52032.33
Human Diseases	Immune disease	59503.81	69803.56	59246.48	61839.77
Human Diseases	Infectious disease: parasitic	42683.45	46098.18	47490.29	39832.27
Human Diseases	Cancer: specific types	43063.22	39260.36	56304.34	35104.08
Human Diseases	Substance dependence	2590.83	24.03	4711.17	457.89
Organismal Systems	Digestive system	7943.82	3907.5	22622.11	6326.37
Organismal Systems	Development and regeneration	1949.11	115.35	4802.86	3465.62
Organismal Systems	Circulatory system	1074.84	8.01	2037.17	211.05
Organismal Systems	Sensory system	10.68	0.632	0.632	0.632

### 3.5 Intestinal metabolites

**Figure 3:**
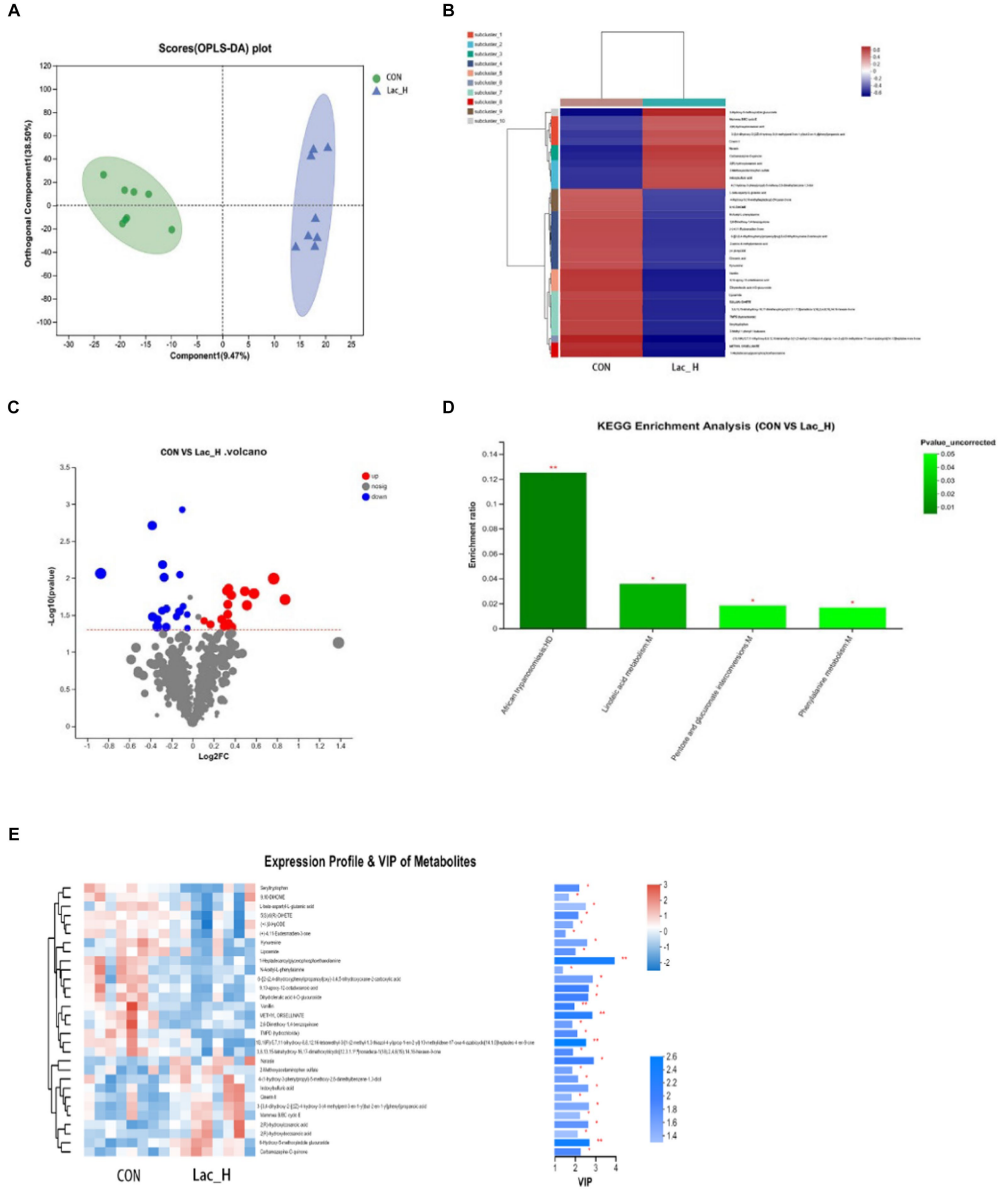
Effects of Lac_H on ileal metabolites of broilers. Multivariate statistical analysis of blank control group and Lac_H group **(A)**. The heat map of cluster analysis of metabolites **(B)**. Volcanic diagram of differentially expressed metabolites **(C)**. Variable importance in projection (VIP) scores of the CON group versus Lac_H group **(D)**. Bubble diagram of metabolic pathway enrichment analysis **(E)**; ^*^, ^**^, and ^***^ represent *p* < 0.05, *p* < 0.01, and *p* < 0.001, respectively.

A total of 6,612 and 5,851 metabolites in ileal contents were determined in positive and negative ion modes, respectively, using LC–MS-based non-targeted metabolomics. A total of 228 metabolites were identified and named based on the HMDB and KEGG databases. Furthermore, orthogonal projection to latent structures-discriminant analysis (OPLS-DA) was employed to select the most predictive and discriminative features to assist classify cation. The loading plot showed a clear separation in metabolites between the Lac_H and CON groups (Figure 3A). The results revealed that the metabolite of broiler significantly changed after treating with *Lactococcus*. Then, the heat map tree of cluster analysis of metabolites (Figure 3B) was constructed, which visualized 50 significantly different metabolites. Overall, there were significant differences in metabolites between the CON and Lac_H groups.

The levels of several metabolites such as 6-hydroxy-5-methoxyindole glucuronide, 3-{3,4-dihydroxy-2-[(2Z)-4-hydroxy-3-(4-methylpent-3-en-1-yl)but-2-en-1-yl]phenyl} propanoic acid, indoxylsulfuric acid, Cinerin II, carbamazepine-*O*-quinone, and 4-(1-hydroxy-3-phenylpropyl)-5-methoxy-2,6-dimethylbenz ene-1,3-diol were upregulated in the Lac_H group, while the levels of 9,10-DiHOME, seryltryptophan, seryltryptophan dihydroferulic acid 4-*O*-glucuronide, 2,6-dimethoxy-1,4-benzoquinone, and kynurenine were downregulated (Figure 3C). Metabolites discriminated among different groups were screened using the variable importance in projection (VIP) scores obtained from the OPLS-DA model, and the ileal contents of metabolic profiles were determined. The metabolites were statistically significant if VIP score ≥ 1 and *p* < 0.05, and *p-*value was calculated by the *t*-test. Metabolites with VIP score > 1.0 and *p* < 0.05 were considered to be significantly influenced by the Lac_H. Thirty significantly affected metabolites were identified in the CON and Lac_H groups, respectively; the top 30 metabolites with the highest VIP scores are presented in Figure 3D.

Metabolic pathway enrichment analysis was performed based on the KEGG database for the differential metabolites between the CON and Lac_H groups, and the metabolic pathway with *p <* 0.05 was significantly enriched for the differential metabolites, including bile secretion, linoleic acid metabolism, drug metabolism-cytochrome P450, phenylalanine metabolism, tryptophan metabolism, and matching metabolites. *Lactococcus* G423 could significantly improve the levels of certain metabolites (6-hydroxy-5-methoxyindole glucuronide, 9,10-DiHOME, *N*-acetyl-l-phenylalanine, and kynurenine), and these metabolites were involved in four metabolic pathways (Table 7). Among them, the pathways of linoleic acid metabolism, phenylalanine metabolism, and pentose and glucuronate interconversions significantly varied (*p* < 0.05) (Figure 3E).

**Table 7:** Effects of *Lactococcus* on the changes in intestinal metabolic pathway in broilers.

First Category	Second Category	Pathway Description	Metabolite	HMDB Superclass	HMDB Class	Pathway_ID
Human Diseases	Infectious disease: parasitic	African trypanosomiasis				map05143
Organismal Systems	Digestive system	Bile secretion	6-Hydroxy-5-methoxyindole glucuronide	Organic oxygen compounds	Organooxygen compounds	map04976
Metabolism	Lipid metabolism	Linoleic acid metabolism	9,10-DiHOME	Lipids and lipid-like molecules	Fatty Acyls	map00591
Metabolism	Amino acid metabolism	Phenylalanine metabolism	*N*-Acetyl-l-phenylalanine	Organic acids and derivatives	Carboxylic acids and derivatives	map00360
Metabolism	Amino acid metabolism	Tryptophan metabolism	Kynurenine	Organic oxygen compounds	Organooxygen compounds	map00380
Metabolism	Carbohydrate metabolism	Pentose and glucuronate interconversions				map00040

### 3.6 Correlation analysis between metabolites and intestinal microbiota

**Figure 4:**
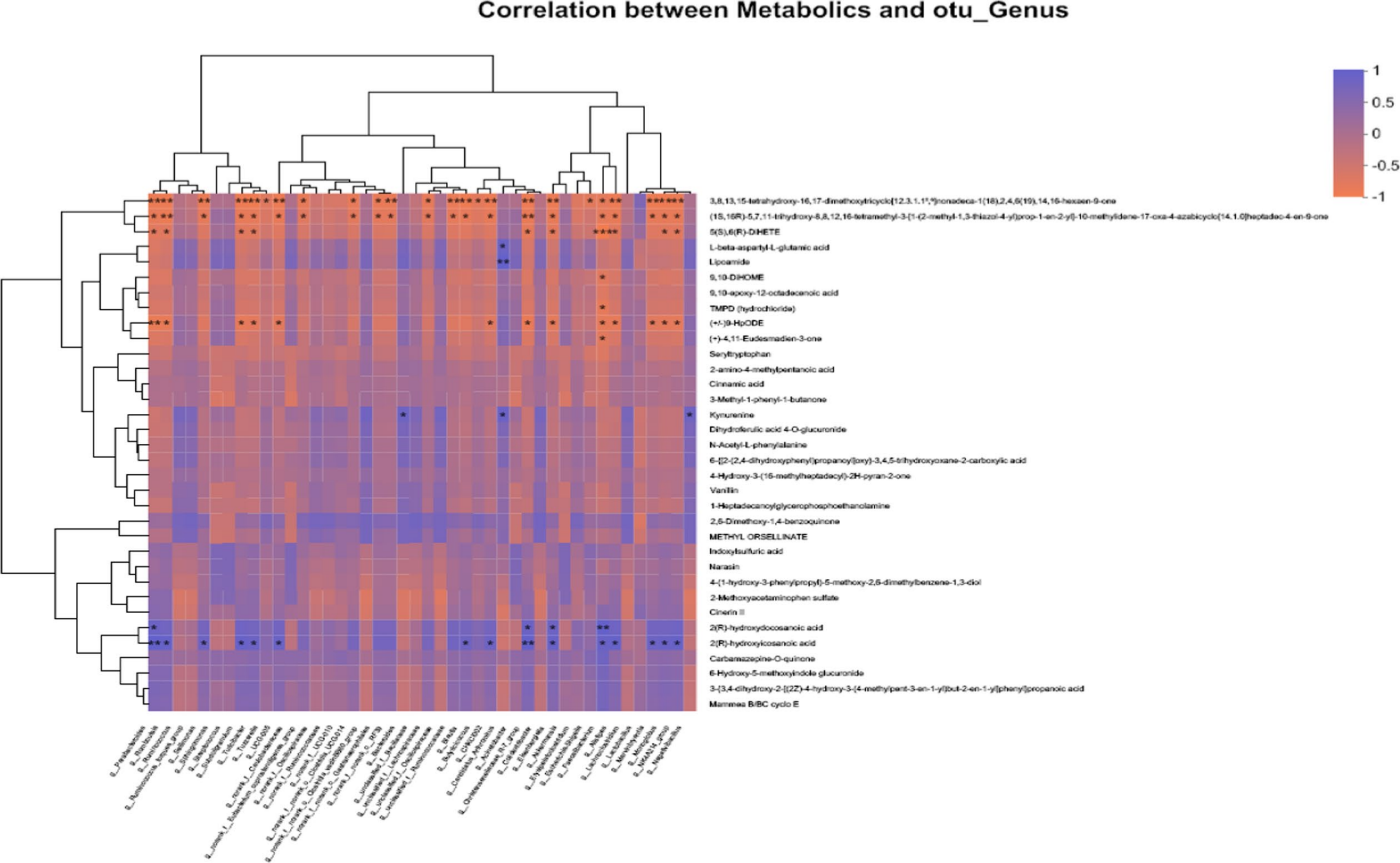
Correlation analysis of “metabolites-intestinal flora” in broilers. Horizontal coordinates indicate metabolites and vertical coordinates indicate gut microbiota; *R* values are shown in different colors in the graph, in which red indicates positive correlation and blue indicates negative correlation; ^*^, ^**^, and ^***^ represent *p* < 0.05, *p* < 0.01, and *p* < 0.001, respectively.

The variations in the intestinal microbiota could be related to the metabolic phenotype. As shown in Figure 4, correlation analysis was performed between 34 different metabolites and 44 bacteria with significantly different relative abundances at the genus level. There was a significant correlation between 2(R)-hydroxyicosanoic acid, 2(R)-hydroxydocosanoic acid, l-beta-aspartyl-l-glutamic acid, 9,10-DiHOME, TMPD (hydrochloride), kynurenine, 6-hydroxy-5-methoxyindole glucuronide, seryltryptophan, and *N*-acetyl-l-phenylalanine and *Parabacteroides, Romboutsia, Sellimonas, Subdoligranulum, Turicibacter, Tuzzerella, Bacteroides, Lachnospiraceae, Butyricicoccus, Candidatus_Arthromitus, Eisenbergiella, Escherichia-Shigella, Faecalibacterium, Alistipes, Marvinbryantia, Monoglobus*, and *Negativibacillus* (all *p* < 0.05).

## 4 Discussion

### 4.1 The effect of *Lactococcus* G423 on growth performance and carcass characteristics in broilers

The diversity and relative abundance of intestinal microbes play an important role in the health of host by participating in metabolism and immunomodulation (Zheng et al., 2021). The findings of the present study suggested that *Lactococcus* G423 could significantly increase ADG in broilers, which were similar to previously reported results (Faseleh et al., 2016; Chen et al., 2018). Supplementation of broilers’ diet with antibiotics could increase body weight gain (Rahman et al., 2012); however, our results showed that Lac_H significantly increase ADG compared with ABX in broiler. This improvement was explained by improved feed conversion efficiency and increased vitality and regulation of the intestinal microflora.

Different from mammals, chickens synthesize fatty acids predominantly in the liver and then export to other tissues including muscle and adipose tissue by the peripheral vascular system. Therefore, the blood lipid index is related to the carcass characteristics. The carcasses from 42-day-old Ross 308 chickens of both sexes, which received the multicomponent probiotics Pro-Biotyk (Em-15) and EMFarma^TM^, did not differ significantly in the percentage of dissected carcass characteristics (Stęczny and Dariusz, 2020). The study by Ding et al. (2021) reveals that lactobacillus reduced abdominal fat deposition in broilers. Our results showed that abdominal fat percentage was lowered by *Lactococcus* G423, and dressing percentage, thigh muscle percentage, and breast muscle percentage had no significant changes among Lac_H, Lac_L, and ABX groups compared with CON groups in broilers. The current results are supported by previous studies on the effect of probiotics on carcasses (Liu et al., 2016; Rybarczyk et al., 2020).

### 4.2 Effect of *Lactococcus* G423 on serum biochemical parameters in broilers

Lipids mainly include triglyceride (TG), phospholipids, and cholesterol (CHO), and the contents of TG and CHO are key indicators of lipid metabolism. The administration of *Paenibacillus polymyxa* up to 0.4 mg/kg diet significantly reduced plasma TC, LDL, and TG (Alagawany et al., 2021).

Ding et al. (2021) found that *Lactobacillus* participated in the lipid metabolism of broilers by reducing the content of TC and TG. Other studies also showed that LAB had effects of blood serum levels TC, HDL-C, LDL-C, and TG on rat (An et al., 2011; Wang et al., 2023). Our results showed that Lac_H significantly decreased the content of TG, TC, and LDL, and Lac_L significantly decreased the content of TG and TC in serum, which was similar to previous studies (Abramowicz, 2019; Abdel-Moneim et al., 2020).

### 4.3 Effect of *Lactococcus* G423 on intestinal microflora in broilers

The gut microbiota community is consisted of diverse types of microbes. In the present study, it was found that *Lactococcus* G423 and ABX altered microbiome diversity in the ileum of broilers and changed the relative abundance rates of *Firmicutes*, *Bacteroidetes*, *Proteobacteria,* and other species. At the phylum level, *Firmicutes*, *Bacteroidetes*, and *Proteobacteria* were the most common phyla in the poultry intestinal samples, which is consistent with the previous findings (Shaufi et al., 2015; Qiao et al., 2018; Zheng et al., 2021). This study indicated that *Firmicutes* was the dominant phylum (>50%) in broilers, and similar results have been previously reported (Danzeisen et al., 2011; Mohd Shaufi et al., 2015). Moreover, this study revealed that the abundance rates of *Firmicutes* were relatively higher in the Lac_L, Lac_H, and ABX groups compared with those in the CON group (*p* < 0.05). Wang et al. (2017) reported that *Lactobacillus* significantly aided in altering the abundance of *Firmicutes* and decreased the content of TG and LDL. *Firmicutes* were associated with lipid metabolites (Turnbaugh et al., 2009). The phylum *Bacteroidetes* has influences on dissolving lipids (Kumar et al., 2018; Chen et al., 2020). *Bacteroides* are also positively correlated with several lipid metabolites (Saxena et al., 2016; Shulpekova et al., 2022). In the present study, it was revealed that the abundance of *Bacteroides* in the Lac_L, Lac_H, and ABX groups was markedly lower than that in the CON group. Meanwhile, considering the TG, LDL, and TC in this experiment, our findings also showed that *Lactococcus* G423 regulated lipid metabolism though regulating intestinal microflora. Although ABX has an effect on the abundance of the *Firmicutes* and *Bacteroides*, there is no effect on the level of LDL, TC, and abdominal fat percentage. Firmicutes and Bacteroidetes can also contribute to host metabolism through several mechanisms, including increased energy harvested from the diet and modulation of lipid metabolism (Greiner and Bäckhed, 2011). Some studies have suggested that a lower abundance of *Bacteroidetes* was associated with increased body weight (Ley et al., 2006; Arumugam et al., 2011). Previous studies also demonstrated that the *Firmicutes/Bacteroidetes* ratio and the growth performance were positively correlated, and this ratio could be indicative of the status of the intestinal bacteria (Haas et al., 2011; Xu et al., 2016). The results of the present study revealed that the *Firmicutes/Bacteroidetes* ratio was relatively higher in the Lac_L, Lac_H, and ABX groups compared with that in the CON group. *Lactococcus* G423 significantly increased ADG by changing the *Firmicutes/Bacteroidetes* ratio. Additionally, the levels of *Proteobacteria* phylum, including some pathogens, such as *Escherichia*, *Salmonella*, *Helicobacter*, and *Vibrio*, were slightly lower in the Lac_L group than those in the CON group, indicating that *Lactococcus* significantly aided in altering the abundance of opportunistic pathogens. However, at the genus level, *Lactobacillus*, *Candidatus_Arthromitus*, *Romboutsia*, and *Bacteroides* were identified as the dominant species in the ileum microbiome. *Lactobacillus*, belonging to the phylum of *Firmicutes*, had markedly higher level in the Lac_L and Lac_H groups than that in the CON group, and the abundance of *Lactobacillus* in the Lac_H group reached the highest rate. *Lactobacillus* is involved in digestive and metabolic processes and in the regulation of local and systemic immune response (Fernández et al., 2016). Moreover, *Lactobacillus* altered lipid metabolism (Wang et al., 2017). Similar effects were observed by Zhou et al. (2016), who studied that *Bacillus licheniformis* and *Lactobacillus* had an effect on the growth and fat deposition in broilers (Gerritsen et al., 2014). *L. fermentum* TSI reduces abdominal fat and improves blood lipid metabolism in HD-induced obese rats (Cho et al., 2020). Therefore, *Lactococcus* G423 significantly aided in altering the abundance of *Lactobacillus*, which participated in gut microbiota, growth, and lipid metabolism in animals (Wu et al., 2019).

*Romboutsia* have been identified in the human gut (Ricaboni et al., 2016), the rat gastrointestinal tract (Gerritsen et al., 2014), and the fecal of hens (Qiao et al., 2018). In the present study, a novel genus *Romboutsia* was found in ileum samples of broilers. However, the abundance of *Romboutsia* was inconsistent among the four groups. Meanwhile, it was revealed that ABX altered the relative abundance rates of other bacteria in ileum contents of broilers, negatively influencing the gut microbiota. Previous studies reported that improper uses of antibiotics have been increased antimicrobial-resistant bacteria as a public health threat (Nhung et al., 2017; Christy et al., 2018; Oniciuc et al., 2018). The results revealed that *Lactobacillus* G423 had more noticeable health benefits compared with antibiotics.

### 4.4 Effect of *Lactococcus* G423 on intestinal metabolites in broiler

The *Lactococcus*-regulated gut microbiota led to alterations in the contents of ileum metabolites. Several significantly altered metabolites were identified in the present study, such as 6-hydroxy-5-methoxyindole glucuronide, 9,10-DiHOME, *N*-acetyl-l-phenylalanine, and kynurenine, which were regulated, and they were involved in four metabolic pathways (Table 7).

Among them, the bile secretion and linoleic acid metabolism were the important metabolic pathways of lipid (Hamilton and Klett, 2021; Shulperkova et al., 2022). It was revealed that 6-hydroxy-5-methoxyindole glucuronide was related to the pathway of bile acid metabolism. Bile acids are also signaling molecules and inflammatory agents that rapidly activate nuclear receptors and cellular signaling pathways, regulating lipid, glucose, and energy metabolism. To a large extent, bile salts are (>95% per cycle) absorbed in the terminal ileum, the final section of the small intestine. The bile salt hydrolase activity has been widely detected in several bacterial genera, including *Bacteroides, Clostridium, Lactobacillus*, and *Bifidobacteria*. In addition, bile acids have been found to affect glucose metabolism by activating FXR and TGR5 receptors, as well as intestinal flora (Jung et al., 2007). *Lactococcus* G423 upregulated 6-hydroxy-5-methoxyindole glucuronide level, suggesting that it may have some regulatory effects on the bile acid metabolism.

Linoleic acid could have beneficial effects on maintaining healthy squabs, as reflected by improved antioxidant capacity and lipid metabolism (Xu et al., 2020). Linoleic acid has shown a correlation with lipid metabolic diseases (Choque et al., 2014). Previous studies have demonstrated that linoleic acid content was associated with probiotics (Hossain et al., 2012; Sahoo et al., 2015). The metabolized product of linoleic acid is 9,10-dihydroxy-12-octadecenoic acid (9,10-DiHOME) (Felipe et al., 2023). More recent research has suggested that DiHOMEs may be important lipid mediators (Hildreth et al., 2020; Zhou et al., 2023). *Propionibacterium acnes* and *Lactobacillus plantarum* have been reported to convert linoleic acid into conjugated linoleic acid (Bo et al., 2017). In the present study, *Lactococcus* G423 downregulated 9,10-DiHOME level, suggesting that it may have some regulatory effects on the linoleic acid metabolism.

*Lactococcus* G423 regulated 9,10-DiHOME and-hydroxy-5-methoxyindole glucuronide by effecting the abundance of *Bacteroides* and *Lactobacillus*, which effected the lipid metabolic pathway of bile secretion and linoleic acid.

## 5 Conclusion

In conclusion, the results of the present study showed that the gut microbiota and the ileum contents of metabolites were significantly correlated, and the metabolites might be considered as mediators in the association between the intestinal microbiota and lipid metabolism. *Lactococcus* G423 could reduce abdominal fat percentage of broilers through the gut microbiota, regulating the pathways of lipid metabolism and bile acid metabolism. *Lactococcus* G423 could ameliorate the lipid metabolism of broilers by integrating the microbiome and metabolome data. Thus, the above-mentioned *Lactococcus* G423 strains can be utilized as a new probiotic combination for animals.

## Data availability statement

The datasets presented in this study can be found in online repositories: https://doi.org/10.6084/m9.figshare.25459783.v2.

## Ethics statement

The animal studies were approved by the HEI Animal Management Certificate No. 11928. The studies were conducted in accordance with the local legislation and institutional requirements. Written informed consent was obtained from the owners for the participation of their animals in this study.

## Author contributions

MW: Funding acquisition, Writing – original draft. WM: Data curation, Writing – review & editing. CW: Conceptualization, Writing – original draft. DL: Resources, Writing – review & editing.

## Footnotes

^1^ http://www.hmdb.ca/

^2^ https://metlin.scripps.edu/

^3^ https://cloud.majorbio.com
