# Supplementary material for: Lactococcus G423 improve growth performance and lipid metabolism of broilers through modulating the gut microbiota and metabolites
Source: Front Microbiol. 2024 Jun 13;15:1381756. doi: 10.3389/fmicb.2024.1381756 (PMC11210191; doi:10.3389/fmicb.2024.1381756)
Supplement: Supplementary file 1 [file Data_Sheet_1.docx]

16sRNA: <https://www.jianguoyun.com/p/DduidFoQwJrjCxiTpI8FIAA>

LC-MC: <https://www.jianguoyun.com/p/DSw2mFsQwZrjCxi9oZIFIAA>

RT-PCR: <https://www.jianguoyun.com/p/DaZQlykQnZ7jCxjCoZIFIAA>

<https://www.jianguoyun.com/p/DXKSqgoQnZ7jCxjFoZIFIAA>
